# Long-term straw returning improve soil K balance and potassium supplying ability under rice and wheat cultivation

**DOI:** 10.1038/s41598-021-01594-8

**Published:** 2021-11-15

**Authors:** Zhiyi Zhang, Dongbi Liu, Maoqian Wu, Ying Xia, Fulin Zhang, Xianpeng Fan

**Affiliations:** National Station for Qianjiang Agro-Environment, Hubei Engineering Research Center for Agricultural Non-Point Source Pollution Control, QianJiang Scientific Observing and Experimental Station of Agro-Environment and Arable Land Conservation, Ministry of Agriculture and Rural Affairs, Key Laboratory of Fertilization From Agricultural Wastes, Ministry of Agriculture and Rural Affairs, Institute of Plant Protection and Soil Fertilizer, Hubei Academy of Agricultural Sciences, Wuhan, 430064 China

**Keywords:** Geochemistry, Fertilization

## Abstract

The aims of the present study were to provide scientific bases for rational use of crop straw to substitute chemical potassium (K) input. The effects of potassium fertilization and straw incorporation on soil K balance and K supplying in a long-term (14 years) field experiment. Five treatments were examined: (1) no fertilization (CK); (2) mineral fertilizing (NPK); (3) straw 6000 kg h m^−2^ (S); (4) NPK with straw 3000 kg h m^−2^ (NPK_1/2_S); and (5) NPK with straw 6000 kg h m^−2^ (NPKS). K composition, K balance and quantity-intensity (Q/I) relationship were studied. Under no fertilization or low straw returned conditions, soil K was unbalanced and deficienct seriously. Straw return at 6000 kg h m^−2^ per season with fertilization improved the soil potassium supply and K balance. Long-term K surplus (4 or 5 years), compared with NPK, the NPKS significantly increased non-special K adsorption (K_nsa_) and non-exchangeable K (K_ne_) by 5.7–11.2 mg kg^−1^ and 65.7–128.1 mg kg^−1^, respectively. Q/I relationship showed cropping without straw K or without fertilizer K resulted in lower quantity (nonspecifically and specifically held K i.e. – ∆K_0_ and K_x_) and intensity (equilibrium activity ratio i.e. CR_0_^K^) of K in tested soils. K-fertilization with straw maintain higher exchangeable K (EK_0_) and a higher difference between EK_0_ and minimum exchangeable K(EK_min_), and would help to prevent depletion in non-exchangeable pool of soil K under intensive cropping. Additionally, The straw return mainly decreased potential buffering capacity for exchangeable pool (PBC^K^_n_), 43.92–48.22% of added K in soil might be converted to exchangeable pool while it was 25.67–29.19% be converted to non-exchangeable pool. The contribution of exchangeable K towards plant K uptake would be higher in the soil with straw than the soil without straw and the non-exchangeable K would be the long-term fixed K as a supplement to the potassium pool. K fertilizer with 6000 kg h m^−2^ straw return in each crop season increased soil available K and slowly available K. The findings underlined importance of the straw return and contribution for sustain K supplying ability of soils.

## Introduction

Potassium (K) is an essential nutrient and plays a particularly crucial role in a number of physiological processes vital to growth, yield, quality, and stress resistance of all crops^1,2^. With the increasing application of nitrogen and phosphorus fertilizers as well as the decrease of organic fertilizer application, large agricultural areas of the world are deficient in K availability, including 3/4 of the paddy soils of China^3,4^. Crop straw not only absorbs K and carries it out of the soils at harvest time, but also an important K fertilizer resource, and retention of crop straw in fields returns a considerable amount of plant K to the soil. As the largest traditional agricultural country in the world, China has a large amount of various crop straws with a yield of 674.91 Mt and retention of crop straw can provide 11.41 Mt of potassium^5^.

The rice–wheat rotation system is one of the largest agricultural production systems, and it covers a total area of ~ 26.7 million hectares (Mha) around the world, including 13.0 Mha for China^6^. Both wheat and rice straws returns are widespread in wheat–rice rotation systems in China because the use of straw returning machines and response to a ban by the Chinese government on field burning of crop straws^7^. The yield and fertility effects of straw return are the focus of agricultural production^8,9^. Yang et al.^12^ reported that ditch-buried rice straw return has the potential to solve the problems of waterlogging stress and that incorporation of total rice straws simultaneously maintains or increases wheat grain yield in the rice–wheat rotation system^10^. A 2-year pot experiment has shown that straw incorporation significantly increases rice yield in most treatments in loamy soil and clay soil (1.6–11.9%)^11^. However, there are also some negative effects on production as a large quantity of straw reduces wheat seedling emergence because more soil pores are created by concentrated straw fragments^12^.

Straw returns to the field can provide potassium needed for growth and improve soil potassium supply capacity and potassium balance. Potassium deficiency is serious in some parts of China, K balances in some areas are as low as − 500 kg K h m^−2^^13^. Long-term potassium deficiency causes the available K decreasing by 21% in a rice–wheat cropping system^14^. Approximately 75–80% of the total K removal is retained in the straw of crops, indicating that retention of crop straw can substantially replenish the K requirement of crops^15^. Yadvinder-Singh et al.^16^ reported that release of K from rice straw increases soil K availability from 50 mg kg^−1^ soil in the untreated control to 66 mg kg^−1^ soil in straw-amended treatments within 10 days after incorporation^16^. Promoting the return of straw to the field has great potential to reduce the use of chemical fertilizer. Yin et al.^5^ reported that straw return to farmlands may counterbalance all of the K_2_O, the majority of the P_2_O_5_, and a portion of the N in chemical fertilizers^5^. K released from maize and rice straw replaces approximately half of the chemical K fertilizer, depending on the available K content in the maize–rice cropping system production^17^. In a rice-rapeseed rotation system, the return straw from the rapeseed season replaces 1/3–2/3 of potash without reducing the yield of rapeseed, and straw return with potassium fertilizer is beneficial to reduce the soil potassium deficiency^18^.

However, the effects of straw return on crop yield, soil fertility and quality have been reported for short-term experiments in rice-rapeseed system, wheat–maize system or rice system, and only a few experiments have been reported using a wheat–rice system^7,19,20^. Thus, a long-term field experiment could demonstrate the effects of straw on crop yield dynamics and soil quality^21,22^. In this study, the dynamic effect of amount of straw return and years on crop yield, soil K, and K balance were investigated by a fixed site field experiment with winter wheat–summer rice rotation for 14 years in the Jianghan Plain. The purpose of this study was to provide scientific bases for rational use of crop straw to substitute chemical K input, to increase crop yield and soil fertility.

## Materials and methods

### Experimental site

The experimental field is located in National Station for Qianjiang Agro-Environment, in Haokou Town (30° 22′ 55.1″ N, 112° 37′ 15.4″ E), Qianjiang City, Hubei Province, China. The permission was obtained from National Station for Qianjiang Agro-Environment. The topography is alluvial plain in nature, featuring the tidal soil-type compost of the river alluvial parent material. This region has a humid subtropical monsoon climate. The annual mean temperature and precipitation are 16.1 °C and 1250 mm, respectively. The basic properties of tested soil at the beginning of the experiment in 2005 are shown in Table 1.

**Table 1:** Selected physicochemical properties of soils studied.

Properties	Value
Particle size analysis (Texture)	Light loam
pH (soil:water = 1:2.5)	7.1
Soil organic matter (g kg^−1^)	20.6
Total N (%)	1.5
Alkali-hydrolyzable N (mg kg^−1^)	121.0
Olsen-P (mg kg^−1^)	19.2
Available K (mg kg^−1^)	59.1
Bulk density (g cm^−3^)	1.2
Smectite (%)	4
HIV and vermiculite (%)	20
Illite (%)	27
Kaolinite (%)	49

HIV, hydroxyl-interlayered minerals.

### Experimental design

The experiment was conducted using a typical winter wheat–summer rice rotation system. Winter wheat was generally planted in early or mid-November after a rotary tillage and was harvested in mid to late May of the following year. Summer rice was planted in early June after a rotary tillage and harvested in late September or early October. The experiment included the following five treatments: (1) CK, wheat and rice were not fertilized during the seasons, and straw incorporation was also not practised; (2) NPK, wheat and rice were only subjected to chemical fertilization in two seasons with no straw return; (3) S, the rice and wheat crops were not applied with chemical fertilizer, and straw return was undertaken at an application rate of 6000 kg h m^−2^ per season; (4) NPK_1/2_S, chemical fertilizer and straw return were undertaken with the amount of fertilizer being the same as under the NPK treatment, and straw return was undertaken at an application rate of 3000 kg h m^−2^ per season; and (5) NPKS, chemical fertilizer and straw return were undertaken with the amount of fertilizer being the same as under the NPK treatment, and straw return was undertaken at an application rate of 6000 kg h m^−2^ per season. All treatments were arranged in a randomized block design with four replicates, and the plot size was 20 m^2^ (5 m × 4 m). The varieties of rice and wheat were Jing Liang You 1377 and Zheng Wheat 9023, purchased from Longping Hi-Tech Seed Industry Co., Ltd and Xiping County Gold Shuo Seed Industry Co., Ltd, respectively. The use of plants in the present study complies with international, national and/or institutional guidelines.

In the fertilizer treatments, the N, P, and K fertilizers were applied at 120, 33, 50 kg h m^−2^ and 150, 39, 75 kg h m^−2^ in the wheat season and rice season, respectively. Each season, 60% of N, total P and K were surface broadcast applied by hand before sowing as a basal fertilization and incorporated into the 0–15 cm soil by rotary tillage, and 40% of N was broadcast applied as topdressing. The topdressing stage occurred during the jointing stage of wheat and tillering stage of rice. The applied fertilizers were urea (46% N), calcium superphosphate (5.2% P), and potassium chloride (50% K).

In each crop season, the crop straw was harvested at ground level, and roots were left in the field. The straw was mixed thoroughly with straw decomposition agent (Wuhan Heyuan Green Organism Co., Ltd., China) after threshing. The straw decomposition agent was mainly composed of typical microbial communities in soils (e.g., bacteria, yeasts, fermenting fungi, and actinomycetes), which were added to facilitate rapid microbial decomposition of the straw for 2–3 weeks. After stacking, the wheat or rice straw was uniformly incorporated into the surface soil by rotary tillage before rice transplantation or sowing of wheat.

### Crop harvest, plant sampling and soil sampling

At annual wheat and rice maturity, each plot was harvested manually, and air-dried grains were weighed. Five rice plants or 50 cm wheat plants in row length were randomly selected from each plot for a separate harvest, and these plants were used for biomass determination. The dry weights of grain and straw were determined after separation and oven drying at 60 °C. For both crops, subsamples of grain and straw were ground and passed through a 0.5 mm sieve for K content determination. An aliquot of air-dried soil samples was passed through a 2 and 0.15 mm sieves.

### Plant and soil chemical analysis

Plant K in grain and straw was digested using the H_2_SO_4_–H_2_O_2_ method. Soil pH was determined by electrode method. Alkaline hydrolysis N was measured using the diffusion method^23^, and available P was determined by the Olsen’s method^24^ in Lu’s publication^23^. Soil available K was extracted using 1 mol L^−1^ ammonium acetate, and water solution K (K_ws_) was extracted using a soil–water ratio of 1:5 for 30 min. Mg(OAc)_2_–K was determined by extraction in 0.5 mol L^−1^ Mg(OAc)_2_. The Mg(OAc)_2_–K consisted of non-special adsorption potassium (K_nsa_) and K_ws_. Special adsorption potassium (K_sa_) was equal to soil available K minus K_nsa_. Non-exchangeable K (K_ne_) was extracted using the hot nitric acid extraction method^25^. All K concentrations were determined with a flame photometer (AAnalyst 400, PerkinElmer, US). SOC was determined by potassium dichromate oxidation at 170–180 °C followed by titration with 0.1 mol L^−1^ ferrous sulphate^23^.

### Quantity/intensity determination

Quantity/intensity (Q/I) study was conducted according to the procedure of Beckett^26^. For each replicated plot, separate samples of 2 g soil were shaken for 30 min with 20 mL of 0.01 mol L^−1^ CaCl_2_ solution having graded concentrations of K (0–2.50 mmol L^−1^) and kept overnight at 25 ± 1 °C for equilibration. After equilibration, the solution was separated by centrifugation and filtration. The K concentrations in the filtrates were determined by emission spectroscopy. The soil was washed with 50% methanol (in water) to remove the entrained 0.01 mol L^−1^ CaCl_2_ solution and extracted with 1 mol L^−1^ NH_4_OAc to get the exchangeable K after equilibration (EK_f_). The filtrate solutions were analyzed for K by flame photometer and Ca, Mg by atomic absorption spectrophotometer (AAS). For a more detailed description, see paper by Islam et al.^27^.

### Clay minerals determination

Organic matter in the soil samples was removed by hydrogen peroxide (30%). Then, the clay (< 2000 nm) fractions were collected by sedimentation according to Stokes' Law. Different clay minerals (< 2000, 450–2000, 100–450, and 25–100 nm) were identified by an oriented X-ray diffractometer (XRD)^28^. The oriented samples were examined using powder XRD analysis (D8 Advance, Bruker, Rheinstetten, Germany) with CuKα radiation (λ = 1.5418 Å) generated at 40 kV and 40 mA. Powder samples were recorded in the range of 5–50°2θ at a scanning speed of 1°2θ min^−1^.

The mean crystal dimension (MCD) was calculated from full width at half maximum height (FWHM) of illite d_001_ using the Scherrer’s equation^29^, and the average layer number (ALN) was obtained by dividing MCD by the d_001_ values of illite. The Scherrer’s equation was ${\text{MCD}} = \frac{K\gamma }{{B\,\cos \theta }},\,\,{\text{where}}$ K was Scherrer constant (0.89 in this study); B was FWHM of d_001_; θ was diffraction angle; γ was X-ray wavelength).

### Calculation

In this study, grain output, crop straw output, potash fertilizer input and crop straw input are discussed as affecting soil potassium pool in the soil–crop system, and K in the atmosphere subsidence and irrigation input are not considered^13,30,31^. The annual straw mulching quantity was strictly controlled, but the annual straw K content was different, resulting in slightly different annual potassium input amount in the S, NPK_1/2_S and NPKS treatments.

Plant K uptake was calculated based on plant K concentration, grain weight and straw weight. The annual soil K budget was calculated using the following equation:

$$ {\text{Soil K balance }}\left( {{\text{kg hm}}^{{ - {2}}} } \right) \, = {\text{ K input }}\left( {{\text{fertilizer K }} + {\text{ straw K}}} \right) \, - {\text{ K removal by crops}} $$

$$ {\text{Relative yield increase }}\left( \% \right) \, = \, ({\text{Yield of NPKS }} - {\text{Yield of NPK}})/{\text{ Yield of NPK}} $$

## Results

### Effect of straw return on K balance

**Figure 1:**
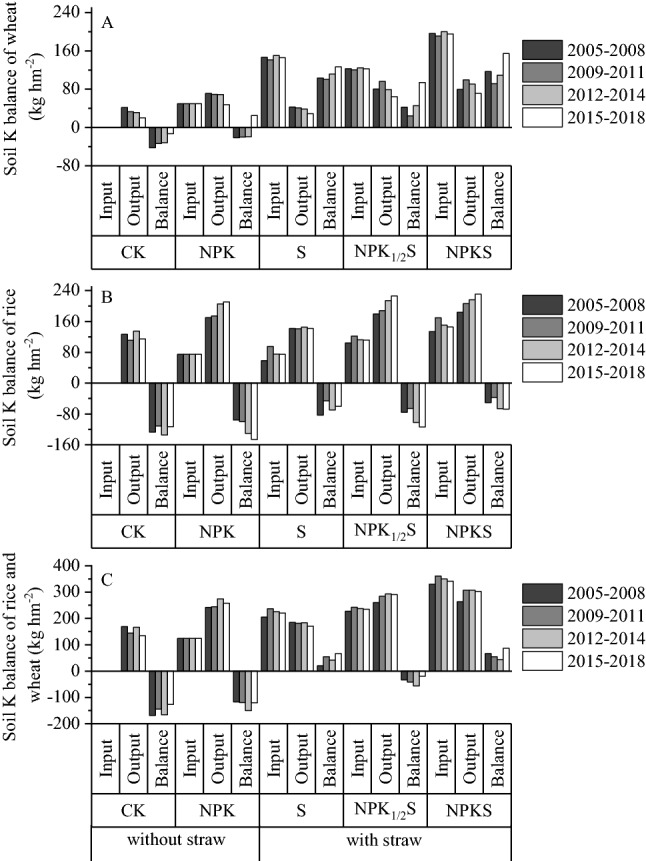
Characteristics of soil potassium balance for different cropping rotation periods from 2005 to 2018. (CK, no fertilization; NPK, mineral fertilizing; S, straw 6000 kg h m^−2^, NPK_1/2_S, NPK with straw 3000 kg h m^−2^ and NPKS, NPK with straw 6000 kg h m^−2^).

The K balance analysis in wheat showed the K was in a deficit state in most no straw return treatments and that the K was in a surplus state in the straw return treatments. The K deficit of the CK treatment (no fertilizer and straw return) was 12.8–42.1 kg h m^−2^ from 2005 to 2018 (Fig. 1A). The K element was close to the balance of input and output in the NPK treatment with approximate K deficits or surpluses by 20 kg h m^−2^. The soil K surplus was between 100.7 and 126.5 kg h m^−2^ in S. After applying straw and fertilizer to the field, the potassium surplus was 24.2–94.3 kg h m^−2^ under a straw return of 3000 kg h m^−2^ and was 91.5–154.8 kg h m^−2^ under a straw return of 6000 kg h m^−2^. These results indicated that a high amount of straw return provided potassium at levels higher than that absorbed by crops, resulting in increased K surplus in the wheat season.

The K balance analysis in rice (Fig. 1B) showed that the K in all treatments was in a deficiency state and that the deficit was much larger in the no or low quantity straw return treatments. The K deficit of the CK treatment ranged from 111.3 to 134.7 kg h m^−2^ and that of the NPK treatment ranged from 94.60 to 146.1 kg h m^−2^. Fertilization alone did not reduce potassium deficiency, but soil K was also in a state of serious deficiency. In addition, the potassium deficiency of the NPK treatment increased with the increase of crop rotation years. Compared with NPK, the soil K deficiency of NPK_1/2_S was slightly reduced with 20.2–32.1 kg h m^−2^. When the amount of straw return was 6000 kg h m^−2^ (NPKS), the soil potassium deficiency was significantly reduced with 45.1–78.5 kg h m^−2^. These findings indicated that high amount of straw return provided the potassium absorbed by crops and slowed down or reduced the potassium deficit in the soil during the rice season.

The annual K balance of the wheat–rice system showed that the potassium balance of CK, NPK and NPK_1/2_S treatment was deficient while that of the S and NPKS treatments was in surplus (Fig. 1C). Under the condition of chemical fertilizer only, the average annual K deficit of wheat–rice rotation was higher, reaching 126.8 kg h m^−2^. Under the condition of a small amount of straw return (3000 kg h m^−2^), the soil potassium balance was slightly deficient with an average annual K deficiency of 37.6 kg h m^−2^. Under the condition of a higher straw return (6000 kg h m^−2^), the soil potassium balance showed a small surplus with an average annual K surplus of 62.8 kg h m^−2^. High straw mulching was beneficial to balance the input and output of potassium literacy, reduce the consumption of soil potassium by crops, alleviate the decrease of soil potassium fertility and maintain the stability of soil potassium fertility.

### Effect of straw return on soil K content

**Figure 2:**
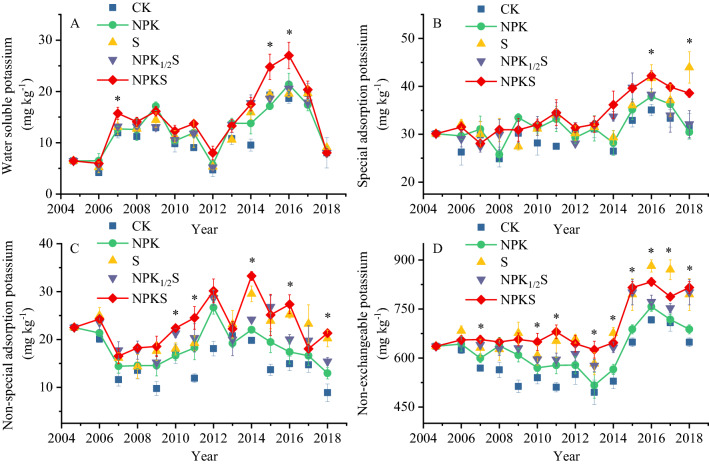
Dynamics of water soluble potassium (**A**), special adsorption potassium (**B**), non-special adsorption potassium (**C**), and non-exchangeable potassium (**D**) from 2005 to 2018. [CK, no fertilization; NPK, mineral fertilizing; S, straw 6000 kg h m^−2^; NPKS, NPK with straw 6000 kg h m^−2^. The significance levels between NPKS and NPK are given (*p < 0.05)].

Over the 14 cropping rotations, K pool was significantly affected by straw incorporation (Fig. 2), the K_ws_, K_sa_, K_nsa_ and K_ne_ contents were higher in NPKS and S treatments than that of NPK and CK treatments in most of investigated years. The content of K_ws_ in NPKS were significantly higher than that of NPK in 3rd, 11th and 12th cropping periods, that of K_sa_ was in 12th and 14th cropping periods. The changes of K_ws_ and K_sa_ were 3.0–7.7 mg kg^−1^ and 4.3–8.2 mg kg^−1^, respectively. In most years after 6th cropping rotations, the K_nsa_ and K_ne_ contents of NPKS treatment were significantly higher than that of NPK, the changes were 5.7–11.2 mg kg^−1^ and 65.7–128.1 mg kg^−1^, respectively. These results indicated that the straw incorporation with K fertilizer increased the amount of available K and direct response of K_nsa_ and K_ne_ content to straw application was larger than that of K_ws_ and K_sa_. The amount of straw application and return years were significantly positive correlation with K balance and K pools from 2005 to 2018 (Table 2).

**Table 2:** Results of ANOVA on the effects of K rate and year and their interactions K balance and K pools from 2005 to 2018.

Effect	K balance	K_ws_	K_nsa_	K_sa_	K_ne_
Straw K rate	465.4***	96.0***	59.9***	12.0**	15.1**
Year	25.3***	51.1***	17.3***	10.6***	46.5***
straw K × Year	0.9^ns^	1.54^ns^	1.5^ns^	1.2^ns^	1.6^ns^

Straw K rate, different amount of straw return, 0, 3000 and 6000 kg h m^−2^ per season; K_ws_, water soluble potassium; K_nsa_, Non-special adsorption potassium; K_sa_, special adsorption potassium; K_ne_, non-exchangeable potassium; F values and significance levels are given (ns *p* > 0.05; *p < 0.05; **p < 0.01; ***p < 0.001).

### Quantity/Intensity relationships (Q/I)

#### Equilibrium K concentration ratio (CR_0_^K^)

The equilibrium K concentration ratio (CR_0_^K^) is presented in Table 3. There was a large variation in CR_0_^K^ in the straw return and no straw return soils. In no straw return soil, CR_0_^K^ were 0.66 and 0.74 × 10^–3^(mol L^−1^)^1/2^ in CK and NPK, respectively. In straw return soil, CR_0_^K^ increased 0.31 and 0.43 × 10^–3^(mol L^−1^)^1/2^ in S and NPKS than CK, respectively. The greatest CR_0_^K^ of 1.09 × 10^–3^(mol L^−1^)^1/2^ was observed in fertilization with straw return 6000 kg h m^−2^ per season.

**Table 3:** Equilibrium K concentration ratio (CR_0_^K^), labile K (K_L_), nonspecifically available K (− ΔK_0_) and specifically available K (K_X_) of rice soil after 14 years of K fertilization.

Treatment	CR_0_^K^ × 10^–3^ (mol L^−1^)^1/2^	K_L_	−ΔK_0_	K_X_
		Cmol kg^−1^		
CK	0.66	0.12	0.086	0.034
NPK	0.74	0.13	0.080	0.050
S	0.97	0.17	0.103	0.067
NPKS	1.09	0.17	0.101	0.069

CK, no fertilization; NPK, mineral fertilizing; S, straw 6000 kg h m^−2^ and NPKS, NPK with straw 6000 kg h m^−2^.

#### Labile K (K_L_)

The K_L_ values in the CK and NPK soil was about 0.12 cmol kg^−1^. Compared with no straw return, the K_L_ values increased about 0.05 cmol kg^−1^ after straw return to soil (Table 3). Positive effect of straw K on − ΔK_0_ contents also could be observed, all straw return treatments showed greater − ΔK_0_ than the treatments without added straw. In addition, the K_X_ of straw return treatments were higher than those without K fertilizer or without straw return. As a result, cropping without straw K or fertilizer K input resulted in lower quantity (nonspecifically and specifically held K i.e. − ΔK_0_ and K_x_) and intensity (equilibrium activity ratio i.e. CR_0_^K^) of K in tested soils.

#### Potential buffering capacities (PBC^K^)

**Figure 3:**
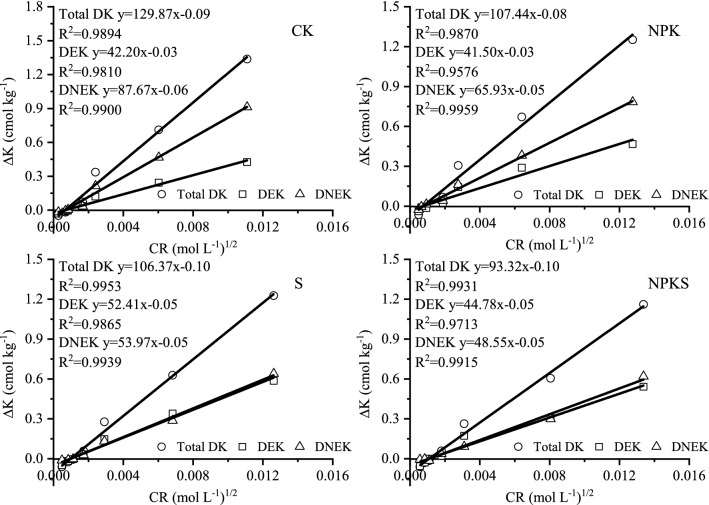
Plots of ΔK versus CR for tested soils with or without straw return. (CK, no fertilization; NPK, mineral fertilizing; S, straw 6000 kg h m^−2^; NPKS, NPK with straw 6000 kg h m^−2^; Total DK or ΔK, amount of total K adsorbed or release from soil during equilibration; CR, potassium concentration ratio, DEK, amount of K adsorbed or release due to exchangeable pool of K during equilibration, DENK, amount of K adsorbed or release due to non-exchangeable pool of K during equilibration).

The tested soils exhibited different capacities for buffering K changes in soil solution system (Fig. 3; Table 4). Potential buffering capacity was higher in CK and NPK soils than S and NPKS soil. In CK soil, the total potential buffering capacity (PBC^K^_t_) was 129.87 cmol kg^−1^/(mol L^−1^)^1/2^. The PBC^K^_t_ of NPK soil was 107.44 cmol kg^−1^/(mol L^−1^)^1/2^ and was decreased with 22.43 cmol kg^−1^/(mol L^−1^)^1/2^. Values of PBC^K^_t_ in S soil was 106.37 cmol kg^−1^/(mol L^−1^)^1/2^ and NPKS soil was 93.32 cmol kg^−1^/(mol L^−1^)^1/2^, dereased by 23.50 and 36.55 cmol kg^−1^/(mol L^−1^)^1/2^ than that of CK, repectively.

**Table 4:** Estimated equilibrium potential buffering capacity of K (PBC^K^) [cmol kg^−1^/(mol L^−1^)^1/2^] of rice soil after 14 year of K fertilization.

Treatment	PBC^K^_t_	PBC^K^_e_	PBC^K^_n_
	cmol kg^−1^/(mol L^−1^)^1/2^		
CK	129.87	42.20	87.67
NPK	107.44	41.50	65.93
S	106.37	52.41	53.97
NPKS	93.32	44.78	48.55

CK, no fertilization; NPK, mineral fertilizing; S, straw 6000 kg h m^−2^ and NPKS, NPK with straw 6000 kg h m^−2^; PBC^K^_e_ = potential buffering capacity due to exchangeable K; PBC^K^_n_ = potential buffering capacity due to non-exchangeable K; PBC^K^_t_ = total potential buffering capacity.

Potential buffering capacity for exchangeable pool (PBC^K^_e_) was lower than the non-exchangeable pool (PBC^K^_n_) in tested soils. The PBC^K^_e_ in no straw return soils were about 42.00 cmol kg^−1^/(mol L^−1^)^1/2^ while the PBC^K^_n_ varied from 65.93 to 87.67 cmol kg^−1^/(mol L^−1^)^1/2^ being the highest in CK. The PBC^K^_e_ in straw return soils varied from 44.78 to 52.41 cmol kg^−1^/(mol L^−1^)^1/2^ being the highest in S while PBC^K^_n_ varied from 48.55 to 53.97 cmol kg^−1^/(mol L^−1^)^1/2^ being the highest in S (Table 4). It indicated the straw return increased a little PBC^K^_e_ and greatly decreased PBC^K^_n_.

#### Equilibrium exchangeable K (EK_0_) and conversion of added K to exchangeable K(ɑ)

**Figure 4:**
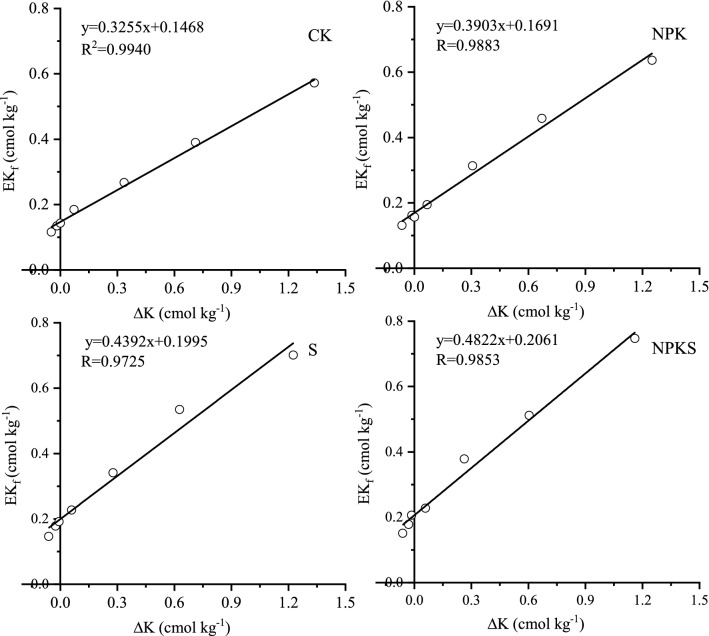
Plots of EK_f_ versus ∆K for tested soils with or without straw return. (CK, no fertilization; NPK, mineral fertilizing; S, straw 6000 kg h m^−2^; NPKS, NPK with straw 6000 kg h m^−2^; EK_f_, NH_4_OAc extractable K determined after equilibration period; ΔK, amount of total K adsorbed or release from soil during equilibration).

Simple significant linear regression equation explained the relationship between EK_f_ and ∆K (the R^2^ of all treatemtns were > 0.97) (Fig. 4). Estimated EK_0_ in unfertilized soil varied from 0.15 to 0.20 cmol kg^−1^, being the highest in S (Table 5). Estimated EK_0_ in fertilized soil varied from 0.17 to 0.21 cmol kg^−1^, being the highest in NPKS. It showed straw return could incread EK_0_ whether in fertilization or unfertilization treatments.

**Table 5:** Effect of long-term straw return on the equilibrium exchangeable K (EK_0_), equilibrium solution K (CK_0_) and magnitude of the conversion of added K to exchangeable K (α) and non-exchangeable K (β).

Treatment	EK_0_ cmol kg^−1^	α %	CK_0_ cmol kg^−1^	β %
CK	0.15	32.55	0.077	37.99
NPK	0.17	39.03	0.088	32.41
S	0.20	43.92	0.093	29.19
NPKS	0.21	48.22	0.102	25.67

CK, no fertilization; NPK, mineral fertilizing; S, straw 6000 kg h m^−2^ and NPKS, NPK with straw 6000 kg h m^−2^.

Slopes of the regression lines for no straw return treatments soil ranged from 0.3255 to 0.3903 and that for straw return soil were 0.4392–0.4822 (Fig. 4; Table 5). This result indicates that 32.55–39.03% of added K in soil might be converted to exchangeable pool while it was 43.92–48.22% in straw return soil (Table 5). It indicated that long-term straw return could bring great change in exchangeable pool of soil K.

#### Equilibrium solution K (CK_0_) and conversion of added potassium to non-exchangeable pool (β)

**Figure 5:**
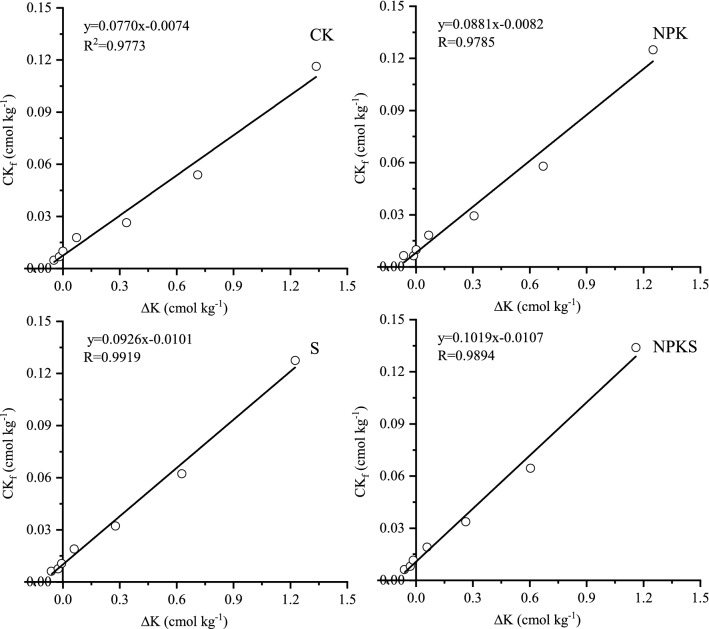
Plots of CK_f_ versus ΔK for tested soils with and without straw return. (CK, no fertilization; NPK, mineral fertilizing; S, straw 6000 kg h m^−2^; NPKS, NPK with straw 6000 kg h m^−2^; CK_f_, Potassium concentration in soil solution after equilibration period; ΔK, amount of total K adsorbed or release from soil during equilibration).

**Figure 6:**
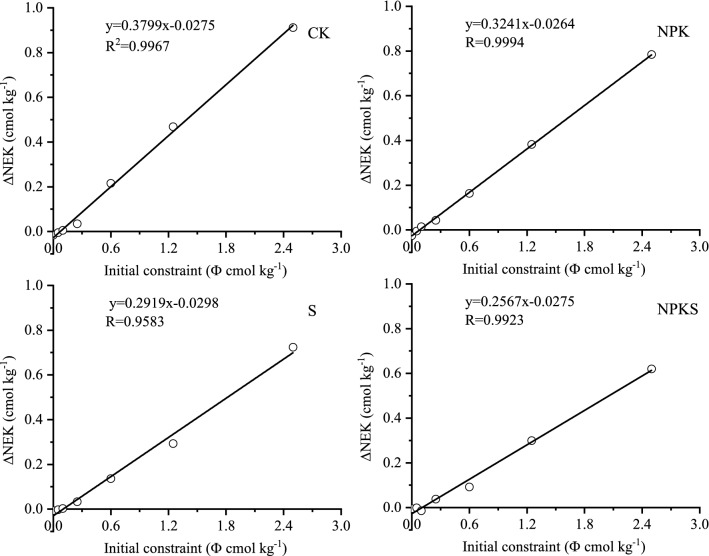
Plots of ∆NEK vs. initial constraint K for tested soils with and without straw return. (CK, no fertilization; NPK, mineral fertilizing; S, straw 6000 kg h m^−2^; NPKS, NPK with straw 6000 kg h m^−2^; ∆NEK, amount of K adsorbed or release due to non-exchangeable pool of K during equilibration).

The relationship between ∆K and K^+^ concentration (CK_f_) in soil solution at different treatments were linear (Fig. 5). The intercepts of different regression lines (CK_0_) varied from 0.077 to 0.102, the straw return treatments were higher than that of no straw return treatments. The amount of K adsorbed or release due to non-exchangeable pool of K during equilibrationcan be described by DNEK and the initial disequilibrium of soil solution applied to the soil can be described by Ф. The significant relationship between DNEK and Ф showed in Fig. 6. The slope for no straw return soil varied from 0.3241 to 0.3799 while for straw return soil varied from 0.2567 to 0.2919 (Fig. 6). These results indicated that 32.41–37.99% of the added K in no straw return soil and 25.67–29.19% in straw return soil would be converted to non-exchangeable pool (Table 5).

#### Critical solution K (CKr) for non-exchangeable K release

**Figure 7:**
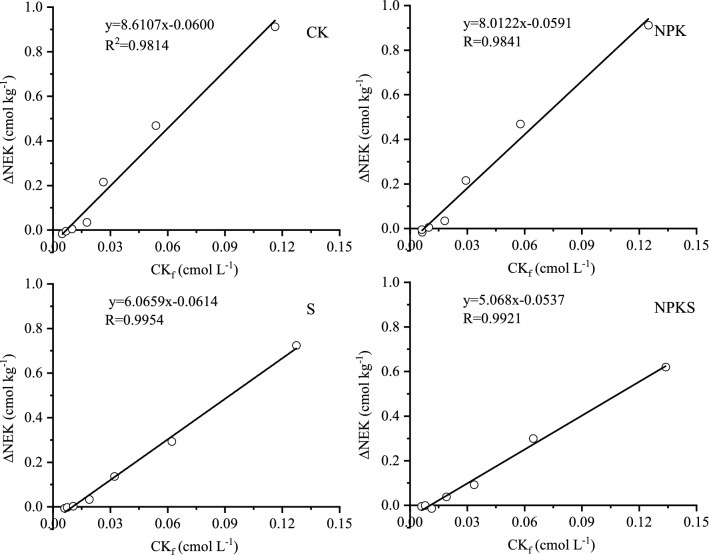
Plots of ∆NEK versus CK_f_ for tested soils with and without straw return. (CK, no fertilization; NPK, mineral fertilizing; S, straw 6000 kg h m^−2^; NPKS, NPK with straw 6000 kg h m^−2^; ∆NEK, amount of K adsorbed or release due to non-exchangeable pool of K during equilibration; CK_f_, Potassium concentration in soil solution after equilibration period).

**Figure 8:**
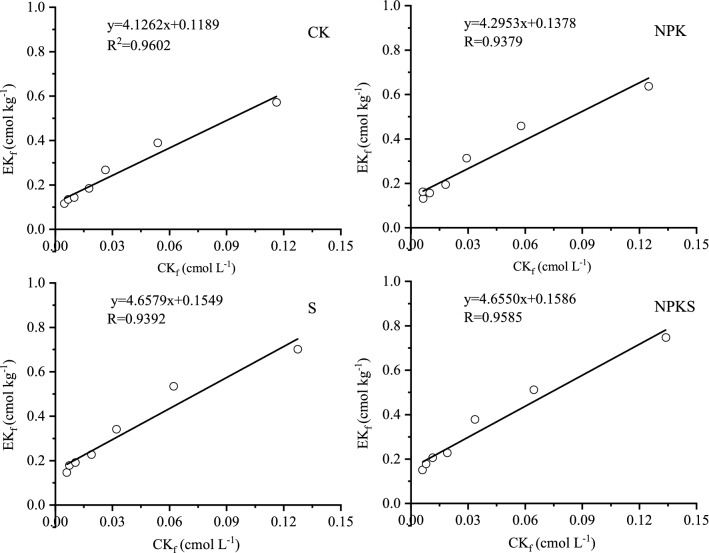
Plots of EK_f_ versus CK_f_ for tested soils with and without straw return. (CK, no fertilization; NPK, mineral fertilizing; S, straw 6000 kg h m^−2^; NPKS, NPK with straw 6000 kg h m^−2^; EK_f_, NH_4_OAc extractable K determined after equilibration period; CK_f_, Potassium concentration in soil solution after equilibration period).

**Figure 9:**
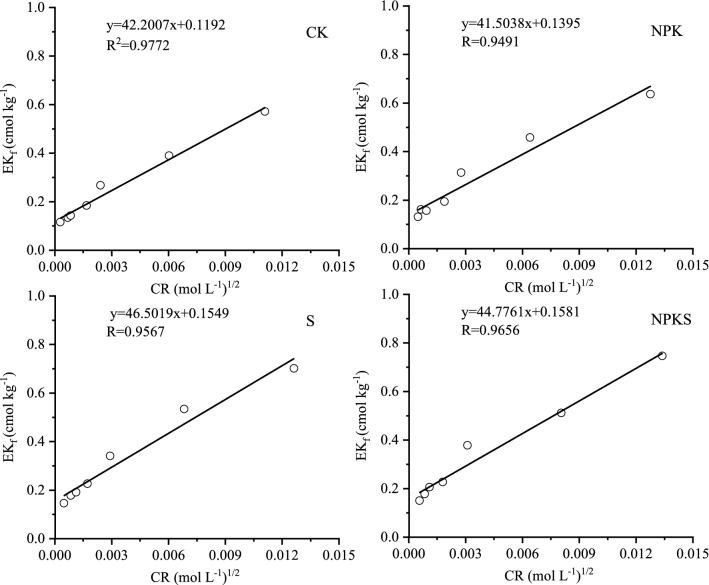
Plots of EK_f_ vs. CR for tested soils with and without straw return. (CK, no fertilization; NPK, mineral fertilizing; S, straw 6000 kg h m^−2^; NPKS, NPK with straw 6000 kg h m^−2^; EK_f_, NH_4_OAc extractable K determined after equilibration period; CR, potassium concentration ratio).

Estimated CK_r_ values for no straw return soil ranged from 0.0070 to 0.0074 cmol L^−1^, however, these values increased with the long term straw return and was 0.0101–0.0106 cmol L^−1^ (Fig. 7; Table 6). The calculated EK_r_ for straw return and no straw return soil were ranged from 0.200 to 0.206 cmol kg^−1^ and 0.147 to 0.169 cmol kg^−1^, respectively (Fig. 8; Table 6). The highest EK_r_ was recorded in fertilization with straw return. Minimum exchangeable K^+^ (E_min_) was derived from the intercepts of Fig. 9. The E_min_ was 0.119–0.139 cmol kg^−1^ in no straw return soil and 0.155–0.158 cmol kg^−1^ in straw return soil. In no straw return soil E_min_ represent about 82% of the EK, while in straw return soil it was about 77% of the EK.

**Table 6:** Effect of long-term K fertilization on the critical value of solution K (CK_r_), exchangeable K (EK_r_) and minimum exchangeable K (E_min_) of rice soil.

Treatment	CK_r_ cmol kg^−1^	EK_r_ cmol kg^−1^	E_min_ cmol kg^−1^	of % EK	EK_0_—E_min_ cmol kg^−1^
CK	0.0070	0.147	0.119	81.20	0.031
NPK	0.0074	0.169	0.139	82.47	0.031
S	0.0101	0.200	0.155	77.65	0.045
NPKS	0.0106	0.206	0.158	76.72	0.052

CK, no fertilization; NPK, mineral fertilizing; S, straw 6000 kg h m^−2^ and NPKS, NPK with straw 6000 kg h m^−2^. CKr, it can be obtained by dividing the intercept by slope of the regression equation of DNEK (in the y-axis) and CKf (x-axis) (Fig. 7); Ekr, it was obtained by interpolation from the relation EK_f_ = f (CK_f_) at CKr (Fig. 8), putting the value of CKr in the place of x, corresponding value of EK_f_ was obtained, which is essentially EKr.

## Discussion

### Change in K balance and soil K pool

According to the Liebig’s nutrient restitution theory, soil potassium balance is the key to the sustainable development of agriculture. In this paper, negative K balances under the CK and NPK treatments indicated continuous depletion of soil K and this situation mainly appeared in rice season (Fig. 1). Under K deficiency, K_ne_ was released and converted into exchangeable potassium for crop absorption^32^. From 2005 to 2014 years, the K_ne_ contents of NPK and CK treatments appeared decreasing trends under K depletion (Fig. 2). Because of release of K_ne_, the content of exchangeable K (K_sa_ and K_nsa_) did not show significant decrease. From 2015 to 2018 years, the wheat yield had a greater extent of reduce, the K balance of wheat was surplus and slow down potassium deficiency of wheat–rice rotation (Fig. 1). The K_ne_ increased about 70 mg kg^−1^ in low yield years (2015–2018 years) (Figs. S1 and S2). Therefore, because of current NPK increased wheat and rice yield than CK, the soil K pool content had a risk of depletion in the rice–wheat rotation when there was an increase in growing years with long term high yield.

Ascribed to the increased K supply of soil due to residue retention, exchangeable K (K_sa_ and K_nsa_) and K_ne_ contents in the straw return treatments increased (Fig. 2). Consistent with the results from our study, Yang et al.^10^ also observed that soil available potassium is significantly improved after straw return^10^. Han et al.^17^ study showed straw return could improve the potential capacity of soil K supplies, straw could be a potential economical K source for crops, and its replenishment efficiency was estimated to be 47% for inorganic K fertilizer under conventional management practices in maize–rice cropping system^17^. Under the condition of surplus potassium, fixation of applied straw K as K_nsa_ and K_ne_ were an important progress of soil potassium cycling (Fig. 2). The K_nsa_ was readily available K for crop, K_ne_ was slowly available K that can release to the soil when soil potassium was in shortage^33,34^. In this study, the changes of K_ne_ (65.7–128.1 mg kg^−1^) was higher than that of K_nsa_ (5.7–11.2 mg kg^−1^), indicating the K mainly fixed as K_ne_. In soils, the K_ne_ was located at lattice wedge sites, interlayer or surface of weathered 2:1 clay mineral which are selective for K ions^35^. After 14 cropping rotations, the semi-quantitative analysis of clay minerals did not show significant difference, but the crystal parameters of illite changed a lot (Table S1). The FWHM and IB of illite in straw return treatments decreased about 0.03, MCD and average layers increased about 10, indicating the surplus K fixed by clay minerals (Table S2). In 14 cropping rotations, the K_ne_ content of NPKS was maintained at about 640 mg kg^−1^ in most years and had amount of increase in low yield years. Therefore, fertilization with 6000 kg h m^−2^ straw return was an important way to improve soil potassium and sustainable soil development.

### Quantity–intensity parameters

#### Equilibrium K concentration ratio (CR_0_^K^)

Q/I curve was used to evaluate the dynamics of K^+^ in straw return and not return soils. The CR_0_^K^ provided a satisfactory estimate of K^+^ availability in soil, the greater CR_0_^K^ values indicate the greater amount of plant available K and a greater K^+^ release into soil solution resulting from a larger pool of soil K^+^^36^. But the plant uptake soil solution K in rice and wheat growth decrease the CR_0_^K^ values in soil. Islam et al.^27^ found that K fertilized soil has the ability to provide more solution K instantly to the growing plants increased CR_0_^K^ values^27^. But in this paper, there was not obvious difference between CK and NPK (Table 3). Althought NPK was fertilized soil, the K balance of NPK was deficit and the CR_0_^K^ of NPK was similar with CK as result. In straw return treatments (S and NPKS), the soil K balance was surplus and CR_0_^K^ increased by about 0.31 × 10^–3^(mol L^−1^)^1/2^. So, soil potassium balance might be an important factor in determining soil CR_0_^K^, straw return had the ability to provide more solution K instantly to the growing plants.

The lower non-specifically available K (− ∆K_0_) values in the CK and NPK treatment were related to depletion of soil K caused by the continuous removal of K with plant biomass. The greater − ∆K_0_ values in the S and NPKS treatments indicated greater release of K into soil solution due to straw return. The higher − ∆K_0_ and CR_0_^K^ in S and NPKS soil was related to the greater accumulation of exchangeable K (Table 3).

#### Potential buffering capacities (PBC^K^)

Higher the PBC^K^, greater depletion of soil K and greater is the ability of a soil to maintain the intensity of soil solution K under changing environments^37,38^. The total potential buffering capacity (PBC^K^_t_) of studied soils was lower in straw return soil than no straw returned soil (Table 4). This finding supported the conclusion from other reports that PBC^K^_t_ of 149 cmol kg^−1^/(mol L^−1^)^1/2^ in non K fertilized soil and of 126–136 cmol kg^−1^/(mol L^−1^)^1/2^ in K fertilized soil^27^. Therefore, increase the input of exogenous potassium would reduce PBC^K^_t_. Lower PBC^K^_t_ of K straw return soil in the present study might also be associated with higher K saturation of this soil compared to K no straw return soil. Roux and Sumner (1968) also reported increase in PBC^K^ with increased K depletion. Removal of adsorbed K from non-specific planner surface sites by cropping increased the buffer capacities, indicating that higher energy sites became involved as the number of cropping increased^37^. The PBC^K^_e_ changed little in different treatments excepted S, while PBC^K^_n_ was lower in straw return soil than no straw returned soil. The results showed that straw potassium existed in the soil in the form of non-exchangeable potassium. The results were also confirmed by the annual evolution of non-exchangeable potassium (Fig. 2).

#### Equilibrium exchangeable K (EK_0_)

Fourteen years of straw return at 6000 kg h m^−2^ each season, increased the EK_0_ of soil. Higher EK_0_ value indicates the greater capacity of soil to supply K to the growing plants. The higher EK_0_ value may have significant importance in arable soils because it can help to maintain proper balance between the solution K and exchangeable K in soil^27^. A soil of higher EK_0_ controls the release of adsorbed K from the exchange sites and result in lower K in soil solution, thus indirectly protecting the soil of a K loss through leaching. Addition of K fertilizer in K-deficient soils increases EK_0_, which in turn results in higher K in soil solution for plant uptake^39^. In this paper, addition of K fertilizer increased EK_0_, which further increased by straw return.

The EK_0_ and EK_min_ had to be considered to assess the effect of straw K and fertilizer on a soil’s K-supplying capacity. If EK_r_ value is close to EK_0_, then it is mostly the K_ne_ pool contributing to plant nutrition^40^. The EK_0_ and EK_min_ of the soils in the present study were different in tested soil (Table 6). So, exchangeable pool of K in the studied soil plays a vital role in K nutrition of rice plant. The EK_min_ is the portion of exchangeable K that is extractable with 1 M NH_4_OAc but would not exchange with Ca^2+^^41^. Even when activity of soil solution K approaches zero, K from EK_min_ portion of exchangeable pool is not released into solution, so it may represent the amount of K^+^ fixed on some clay interlayer sites and is almost unavailable to plants^27^. Hence, the difference between EK_0_ and EK_min_ would indicate the plant available part of exchangeable K pool in soil^2^. In the present study, the differences between EK_0_ and EK_min_ were higher in straw return soil than no straw return soil, indicating that contribution of exchangeable K towards plant K uptake would be higher in the soil with straw than the soil without straw. Application of straw and K fertilizer was able to maintain higher value of the difference between EK_0_ and EK_min_ than other treatments (Table 6). Therefore, K-fertilization with straw maintain higher EK_0_ and a higher difference between EK_0_ and EK_min_, and would help to prevent depletion in non-exchangeable pool of soil K under intensive cropping. Such findings clearly highlight the importance of adequate K input through fertilizer with straw on reducing the contribution of soil’s nonexchangeable pool towards plant K nutrition.

### Conversion of added potassium to non-exchangeable pool (β)

Like seen for ɑ, K fertilization could not bring great change in β, but K fertilization with straw return increased ɑ and decreased β. The studied soil had larger ɑ than β in straw return soil, much of the applied K (fertilizer K and straw K) converted to K_ne_ in straw return soils (Table 5). The impact of the exchangeable and non-exchangeable pools on K^+^ dynamics in the soil solution system could be indicated though the slope (β) between the ∆NEK and the initial constrain indicates^35^. The larger the β the greater the portion of added K^+^ converted to K_ne_ (fixed) at positive Ф or the more fixed K^+^ released at negative Ф^42^. Thus, it can be expected that when K^+^ fertilizer and straw are applied to a soil having a large ɑ and a small β, much of the K^+^ is held as exchangeable and would be available to plants. But in K fertilizer without straw, K fertilizer was applied to a less K supplying soil, K^+^ is held as nonexchangeable, would be available to plants after releasing the short-term fixed K.

## Conclusions

The K deficit of the rice–wheat rotation was 126.8 kg h m^−2^ in NPK, and the K deficit mainly appeared in the rice season. High straw return (6000 kg h m^−2^ per season) was beneficial to balance the input and output of potassium to achieve an annual potassium surplus by 62.8 kg h m^−2^.

The straw incorporation with K fertilizer increased the amount of available K and direct response of K_nsa_ and K_ne_ content to straw application was larger than that of K_ws_ and K_sa_, The amount of straw application and return years were significantly positive correlation with K balance and K pools from 2005 to 2018.

The cropping with straw K and fertilizer K input resulted in higher quantity (nonspecifically and specifically held K i.e. − ΔK_0_ and Ks) and intensity (equilibrium activity ratio i.e. CR_0_^K^) of K in tested soils.The greatest CR_0_^K^ of 1.09 × 10^–3^(mol L^−1^)^1/2^, − ΔK_0_ of 0.101 cmol kg^−1^ and K_X_ of 0.069 cmol kg^−1^ were observed in fertilization with straw return 6000 kg h m^−2^ per season.

The straw return increased a little PBC^K^_e_ and greatly decreased PBC^K^_n_, 43.92% to 48.22% of added K in soil might be converted to exchangeable pool while it was 25.67–29.19% be converted to non-exchangeable pool. The contribution of exchangeable K towards plant K uptake would be higher in the soil with straw than the soil without straw and the non-exchangeable K could be long-term fixed.

The significant relationship between DNEK and Ф indicated that 32.41–37.99% of the added K in no straw return soil and 25.67–29.19% in straw return soil would be converted to non-exchangeable pool. Addition of K fertilizer increased EK_0_, which further increased by return straw, fertilization with straw maintain would help to prevent depletion in non-exchangeable pool of soil K under intensive cropping.

When K^+^ fertilizer and straw are applied to a soil, much of the K^+^ is held as exchangeable and would be available to plants. But in K fertilizer without straw, K fertilizer was applied to a less K supplying soil, K^+^ is held as nonexchangeable, would be available to plants after releasing the short-term fixed K.

## Supplementary Information

Supplementary Information.
