# Supplementary material for: Long-term straw returning improve soil K balance and potassium supplying ability under rice and wheat cultivation
Source: Sci Rep. 2021 Nov 15;11:22260. doi: 10.1038/s41598-021-01594-8 (PMC8593129; doi:10.1038/s41598-021-01594-8)
Supplement: Supplementary file 1 — Supplementary Information. [file 41598_2021_1594_MOESM1_ESM.docx]

## Figure and Tables

**Fig. S1** Wheat yields as affected by different straw return treatments from 2005 to 2018 (CK, no fertilization; NPK, mineral fertilizing; S, straw 6000 kg hm^-2^, NPK_1/2_S, NPK with straw 3000 kg hm^-2^ and NPKS, NPK with straw 6000 kg hm^-2^. * indicates significant differences among NPK and NPKS treatments (*p* < 0.05), and error bar values represent the mean±SD. The solid and short dash lines represent the median and mean values, respectively. The lower and upper edges represent the 25th and 75th percentiles of all data, respectively. The bars and dots represent the 5th and 95th percentile of all data, respectively. The inside or outside of the boxes represent the <5th and >95th percentiles of all data, respectively.)

**Fig. S2** Rice yields as affected by different straw return treatments from 2005 to 2018 (CK, no fertilization; NPK, mineral fertilizing; S, straw 6000 kg hm^-2^, NPK_1/2_S, NPK with straw 3000 kg hm^-2^ and NPKS, NPK with straw 6000 kg hm^-2^. * indicates significant differences among NPK and NPKS treatments (*p* < 0.05), and error bar values represent the mean±SD. The solid and short dash lines represent the median and mean values, respectively. The lower and upper edges represent the 25th and 75th percentiles of all data, respectively. The bars and dots represent the 5th and 95th percentile of all data, respectively. The inside or outside of the boxes represent the <5th and >95th percentiles of all data, respectively.)

**Table S1**

Semi-quantitative analysis of clay minerals by XRD techniques using an oriented specimen slide (%)

| Treatment | Smectite | Vermiculite and HIV | Illite | Kaolinite |
| --- | --- | --- | --- | --- |
| CK | 4.9^a^ | 19.1 | 26.0 | 50.1 |
| NPK | 4.5 | 19.0 | 29.0 | 47.4 |
| S | 3.0 | 19.9 | 28.5 | 48.6 |
| NPKS | 3.2 | 21.5 | 25.1 | 50.2 |

^a^ Semi-quantitative analysis of Mg-gly XRD patterns; HIV, hydroxyl-interlayered minerals; CK, no fertilization; NPK, mineral fertilizing; S, straw 6000 kg hm^-2^ and NPKS, NPK with straw 6000 kg hm^-2^.

**Table S2**

Some properties of illite of tested soils

| Treatment | d-value (Å) | FWHM (º2θ) | IB | MCD (Å) | ALN |
| --- | --- | --- | --- | --- | --- |
| CK | 0.997 | 0.280 | 0.406 | 105 | 106 |
| NPK | 0.997 | 0.281 | 0.411 | 104 | 105 |
| S | 0.997 | 0.252 | 0.384 | 116 | 117 |
| NPKS | 0.997 | 0.250 | 0.381 | 117 | 118 |

FWHM, Full width at half maximum height of 001 diffraction peak; IB, integral width of 001 diffraction peak; MCD, mean crystal dimension along 001 direction; ALN, average layer number; CK, no fertilization; NPK, mineral fertilizing; S, straw 6000 kg hm^-2^ and NPKS, NPK with straw 6000 kg hm^-2^.
